# Repetitive transcranial magnetic stimulation paired with a 3D-printed ankle-foot orthosis for ankle-foot dysfunction among older adults following subacute stroke: clinical findings and exploratory neurophysiological analyses

**DOI:** 10.3389/fneur.2026.1859162

**Published:** 2026-10-13

**Authors:** Yuting Zhang, Hehe Chen, Rui Xu, Lingyu Zhu, Siyuan Song, Weixin Zhu

**Affiliations:** 1 Jinhua Municipal Central Hospital, Jinhua, Zhejiang, China; 2 Department of Anesthesiology, Montefiore Medical Center, Albert Einstein College of Medicine, Bronx, NY, United States

**Keywords:** 3D- printed ankle-foot orthosis, ankle-foot dysfunction, corticospinal excitability, older adults, repetitive transcranial magnetic stimulation, stroke

## Abstract

**Objective:**

This work evaluated clinical effects of repetitive transcranial magnetic stimulation (rTMS) plus custom-made 3D-printed ankle-foot orthosis (AFO) for older adults living with post-stroke ankle-foot impairment. Neurophysiological assessments were also carried out for exploratory purposes, drawing exclusively on indirect physiological readouts.

**Materials and methods:**

Sixty older participants with ankle-foot dysfunction after subacute stroke were randomized into three parallel groups: conventional-only rehabilitation, 3D-printed AFO therapy, and combined rTMS-AFO intervention (*n* = 20 per group). Evaluations including ankle active range-of-motion (AROM), resting motor threshold (RMT), integrated surface electromyography (iEMG), Clinical Spasticity Index (CSI), Fugl-Meyer Assessment of the Lower Extremity (FMA-LE), Berg Balance Scale (BBS), and 10-Metre Walking Test (10MWT) were performed at baseline and four-week post-intervention time-point.

**Results:**

Noticeable within-group functional improvements emerged in all three study arms upon completion of treatment (*p* < 0.001). Participants assigned to combined therapy achieved greater functional improvements compared with those receiving standalone 3D-printed-AFO or conventional rehabilitation (*p* < 0.05). The conventional rehabilitation group produced the weakest magnitude of functional recovery across measured endpoints.

**Conclusion:**

Compared with single-modality rehabilitation approaches, adding rTMS to 3D-printed AFO therapy brought measurable improvements in ankle mobility, muscle recruitment, balance and walking performance. These clinical benefits occurred alongside elevated excitability within the ipsilesional corticospinal tract. Explanations regarding sensorimotor integration and afferent-efferent circuit regulation should be regarded as exploratory hypotheses because direct neural measurements were not obtained in this investigation. Further independent research is needed to test these mechanistic assumptions.

## 1 Introduction

Stroke represents a worldwide major source of mortality and persistent disability, imposing substantial burdens on patients, family caregivers and public-health systems. Within China and many other regions, it ranks among the top conditions that produce physical disability in older populations (1). Ankle-foot dysfunction represents one of the most prevalent and disabling sequelae following stroke. Typical manifestations include foot drop, varus deformity, exaggerated muscle spasticity, and pathological gait patterns, which severely compromise walking ability, balance control, and independent daily functioning (2). Even though multiple rehabilitation strategies exist, isolated interventions frequently deliver modest gains, given that they seldom tackle central motor deficits and peripheral biomechanical abnormalities at the same time.

Repetitive transcranial magnetic stimulation (rTMS) is a non-invasive neuromodulation technique (3) and can alter cortical excitability (4), improve corticospinal transmission, and promote use-dependent plasticity (5). It boosts central efferent drive and improves voluntary motor control of ankle-foot musculature (6). Still, rTMS cannot directly correct established ankle-foot deformities. For this reason, abnormal stance-phase gait and balance instability may persist, slowing observable functional recovery (7).

Ankle-foot orthosis (AFO) serves as a core peripheral intervention for correcting ankle-foot deformities and optimizing gait after stroke. Traditional AFOs are mostly mass-produced in a standardized fashion, with limitations including poor fitting, excessive weight, inadequate ventilation, and uneven pressure distribution across the foot and ankle. These issues contribute to low wearing compliance, elevated risk of pressure injury, and failure to meet individualized biomechanical needs (8). Thanks to advances in digital scanning, precision manufacturing, and rapid prototyping (9), 3D-printed AFOs can be made to fit each patient. They are lightweight and well-fitting. Clinical observations from our centre indicate these orthoses offer solid mechanical support, assist deformity correction, optimize gait and cut down compensatory movement patterns. Improved mechanical alignment with a 3D-printed AFO may enhance proprioceptive feedback from the ankle to the central nervous system, which in turn supports motor accuracy and cortical plasticity (10).

Only a small number of rehabilitation studies have combined central neuromodulation and custom-tailored peripheral biomechanical correction into one treatment paradigm. We raise an exploratory hypothesis: combining rTMS and 3D-printed AFO may yield therapeutic gains greater than simple additive effects and potentially build a sensorimotor closed-loop rehabilitation pathway (11). Under this conceptual framework, rTMS raises cortical excitability and descending motor output (12), while custom-built 3D-printed AFO supplies movement stability and continuous proprioceptive afferent signals. Such peripheral sensory input might in theory strengthen use-dependent cortical plasticity and gradually refine voluntary motor performance. It is worth stressing that this mechanistic model stays speculative and was not empirically verified in our trial (13).

This three-arm randomized controlled trial had two predefined primary objectives. First, to compare clinical functional results from three groups. Outcome domains covered ankle-foot mobility, motor recovery, balance and walking performance for older adults in the subacute stroke period. The second objective involved exploratory neurophysiological monitoring; RMT and iEMG acted as indirect surrogate markers to capture changes in ipsilesional corticospinal excitability and peri-ankle muscle activation. Separated from these pre-set objectives, we proposed an exploratory hypothesis: add-on high-frequency rTMS targeting ipsilesional M1, combined with peripheral support from a 3D-printed AFO, may bring extra rehabilitation benefits correlated with raised ipsilesional corticospinal excitability. Results from this study provide observational clinical evidence and preliminary neurophysiological background for refining combined central-peripheral rehabilitation strategies targeting post-stroke ankle-foot dysfunction among older patients.

## 2 Materials and methods

### 2.1 Ethical approval

This study received ethical approval from the Medical Ethics Committee of Jinhua Municipal Central Hospital (Ethics approval number: 20242080101). The study was conducted in strict adherence to the Declaration of Helsinki. All participants provided written informed consent prior to study enrollment. This randomized controlled trial was retrospectively registered in the Chinese Clinical Trial Registry (ChiCTR2600121992; registered April 2026). Participant recruitment and full-set data collection were finished between June 2025 and December 2025. Pre-enrolment prospective trial registration was not completed at study commencement, which stemmed from insufficient team awareness of ICMJE-CONSORT reporting standards for early-phase trial planning. Pre-defined primary and secondary outcome endpoints are documented in registry files. The prespecified primary outcome was ankle active range-of-motion; secondary outcomes included resting motor threshold (RMT), integrated surface electromyography (iEMG), Clinical Spasticity Index (CSI), Fugl-Meyer Assessment of the Lower Extremity (FMA-LE), Berg Balance Scale (BBS), and 10-meter walking test (10MWT).

### 2.2 Study participants and inclusion/exclusion criteria

Sample-size calculation was completed in PASS 15.0 software based on a one-way ANOVA model, with ankle active dorsiflexion AROM as the primary clinical endpoint. Input parameters were sourced from existing rehabilitation research focusing on ankle functional recovery after stroke. We assumed a between-group mean difference of 2.20° and pooled standard deviation of 2.10, corresponding to a Cohen’s *f* of 0.52. Two-sided α was set to 0.05 and statistical power (1-*β*) set to 0.80. Calculations suggested a minimum sample of 17 subjects for each study arm. Taking the expected 15% dropout rate into account, we aimed to recruit 20 participants per group (17 ÷ [1–0.15] ≈ 20), for a total planned sample of 60 subjects. This study enrolled 60 elderly inpatients with post-stroke ankle-foot dysfunction (foot drop and foot varus) who were hospitalized in our hospital from June to December 2025. An investigator independent of participant recruitment, outcome assessment and treatment generated random allocation sequences using a random-number table. Group-assignment slips were sealed inside sequentially numbered, opaque sealed envelopes. Envelopes were only opened after participants gave written informed consent and satisfied all eligibility criteria, so as to guarantee allocation concealment and reduce selection bias. Screening investigators had no advance access to randomization outputs. Subjects were randomly divided into the conventional group, the 3D-printed group and the combined group by random number table, with 20 cases in each group (Figure 1).

**Figure 1:**
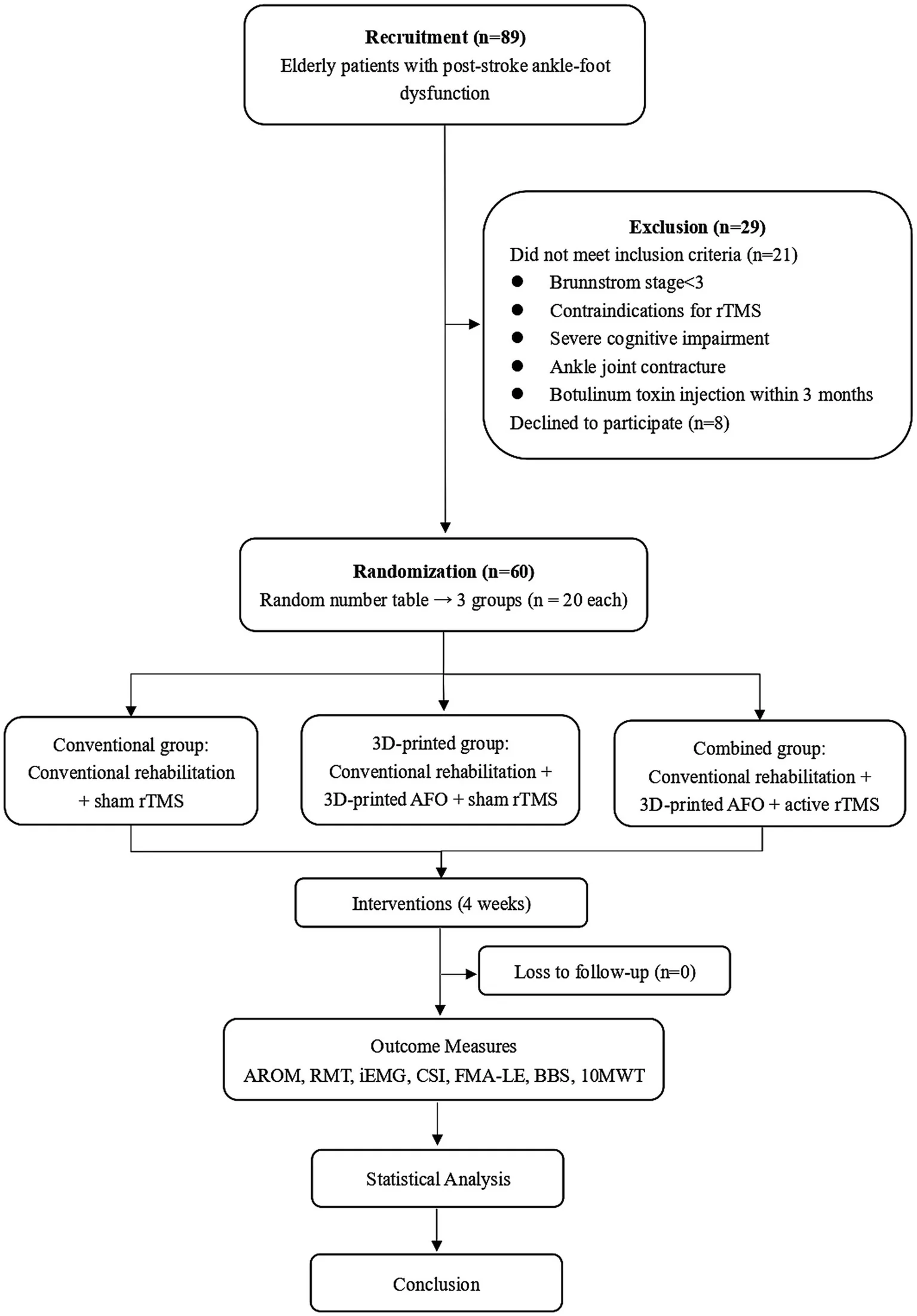
Flowchart of participant recruitment, grouping, and intervention.

Inclusion criteria: (1) first-onset stroke, confirmed as cerebral infarction or intracerebral hemorrhage by cranial CT/MRI, meeting the diagnostic criteria for cerebrovascular disease (14); (2) age 60–80 years, disease duration 1–6 months; (3) presented with foot drop and foot varus gait, Brunnstrom stage ≥3 in the hemiplegic lower extremity, Clinical Spasticity Index (CSI) ≤ 12, standing balance grade 3; (4) hemodynamically stable, GCS score ≥13, able to walk ≥10 m independently or under supervision; (5) good compliance and able to cooperate fully throughout the study.

Exclusion criteria: (1) implanted metallic devices such as pacemakers or cardiac stents; (2) history of epileptic seizures or high seizure susceptibility (15); (3) hemorrhagic transformation; (4) dysfunction attributable to spinal cord injury, peripheral neuropathy, lower extremity fracture, joint contracture, etc.; (5) received botulinum toxin injection or antispastic medication within the preceding 3 months; (6) severe visual, auditory, or cognitive impairment; (7) allergy to 3D-printing materials or intolerance to AFO; (8) acute stroke (onset <3 weeks).

### 2.3 Intervention protocols

All patients received standard post-stroke medical management, including medications to support cerebral circulation, neuroprotection, antiplatelet therapy, antioxidant treatment, and control of blood pressure and blood glucose. All patients underwent standardized rehabilitation training administered by uniformly trained therapists, including proper limb positioning, passive, active, and resistive exercises of the affected limb, with emphasis on ankle dorsiflexion and eversion training. Balance training was gradually progressed to weight shift, gait correction, and walking training (16), 30 min per session, once daily, 5 days per week. Physical therapy included neuromuscular electrical stimulation (NMES) of the tibialis anterior muscle on the affected side, 20 min per session, once daily, 5 days per week. The intervention lasted for 4 weeks (17).

- Conventional group: conventional rehabilitation + sham AFO + sham rTMS
- 3D-printed group: conventional rehabilitation + 3D-printed AFO + sham rTMS
- Combined group: conventional rehabilitation + 3D-printed AFO + active rTMS

The preparation, assembly procedures and wearing duration of the 3D-printed AFO were described as follows. A high-precision white-light 3D scanner (EinScan-Pro 2X, Shining 3D, China) was adopted to acquire surface data of the affected foot, with a single-scan acquisition time ≤ 90 s. Raw scanned point-cloud data were imported into reverse-modeling software Geomagic Wrap 2023 for processing. The preset correction angle for ankle dorsiflexion was 10°, and subtalar joint eversion correction was set at 5°. The orthosis was fabricated via fused deposition modeling (FDM) using medical-grade carbon-fiber-reinforced PLA material. The finished orthosis was double-calibrated by a professional orthotist under weight-bearing stance and dynamic gait conditions to ensure effective correction of foot-inversion and foot-drop deformities. Patients in the 3D-printed group and combined group wore the personalized 3D-printed AFO throughout rehabilitation training over the 4-week intervention period, with a cumulative daily wearing time of ≥ 6 h. Rehabilitation therapists recorded wearing compliance on-site each day. All participants completed the full-course intervention, and no AFO-related skin injury or intolerance-induced detachment occurred. Patients in the conventional group were provided with placebo sham orthoses that perfectly matched the personalized 3D-printed AFO in appearance, weight and outer contour. All rigid mechanical support structures were removed from the sham orthoses, yielding no corrective effect on ankle deformities. The sham orthoses were used merely for blinding control to eliminate confounding bias associated with orthosis wearing, with identical wearing-time requirements as those for the 3D-printed group and combined group.

In this trial, a standard TMS stimulator equipped with an-8-figure coil manufactured by Jiangxi Brain Regulation Company was used. Based on the international 10–20 EEG positioning system for anatomical navigation, the tibialis anterior muscle was selected as the target muscle to localize the motor hotspot of the lower-limb representation in the ipsilesional primary motor cortex (M1). The EMG recording electrode was placed over the muscle belly of the tibialis anterior, the reference electrode was attached to the medial malleolus, and the ground electrode was fixed over the middle segment of the fibula. Formal rTMS treatment was delivered at a frequency of 10 Hz with stimulus intensity set at 80–120% of the resting motor threshold (RMT). Each 20-min rTMS session adopted a cyclic stimulation paradigm: 2-s stimulation trains at 10 Hz followed by an inter-train interval of 8 s. A total of 120 trains were delivered, generating 2,400 valid pulses per session. For real stimulation, the 8-figure coil was placed tangentially against the scalp, with the coil center aligned to the marked motor hotspot. An adjustable cantilever arm provided constant mild pressure to secure the coil and prevent displacement during treatment. For sham stimulation, the figure-8 coil was positioned vertically against the participant’s scalp; it produced identical acoustic noise without delivering effective neuromodulation. The intervention was administered once daily, 5 days per week, for a continuous period of 4 weeks. Stimulation intensity was individually adjusted for each participant within the window of 80-120% of each subject’s measured RMT. During the 4-week intervention course, clinicians reassessed RMT periodically. Where RMT decreased by > 5% (indicating elevated cortical excitability), stimulus intensity was moderately reduced to avoid potential cortical over-excitation; intensity remained capped at 120% RMT if RMT showed little measurable change.

### 2.4 Outcome measures

All assessments were performed by blinded evaluators at baseline and after 4 weeks of intervention.

#### 2.4.1 Primary outcome measure: ankle active range of motion (AROM)

With the patient supine and the knee extended, the active ranges of ankle dorsiflexion and plantarflexion were measured using a standard goniometer.

#### 2.4.2 Secondary outcome measures

##### 2.4.2.1 Electrophysiological secondary indicators

1. Motor cortex excitability (MCE)
  Motor cortex excitability, quantified via resting motor threshold (RMT; abbreviated MCE within tables and figures). A lower RMT signifies higher cortical excitability (18).
2. Surface electromyography assessment
  The integrated electromyography (iEMG) values of the tibialis anterior during ankle dorsiflexion and the peroneus longus, peroneus brevis, medial gastrocnemius, and lateral gastrocnemius during ankle plantarflexion were recorded. The mean value of three measurements was calculated (19). iEMG serves as an indicator of muscle contraction capacity and shows positive correlation with motor-function performance.

##### 2.4.2.2 Clinical functional secondary indicators

1. Spasticity assessment
  Spasticity was assessed using the CSI, encompassing tendon reflexes, muscle tone, and clonus. Scores were graded as: 0–9 mild, 10–12 moderate, and 13–16 severe (20).
2. Fugl-Meyer assessment of the lower extremity (FMA-LE)
  The lower-extremity FMA-LE contains 17 items, each scored 0–2 points, with a total score of 0–34. Higher scores denote better motor function.
3. Balance function
  Balance function was evaluated using the Berg Balance Scale (BBS), which includes 14 items scored on a 5-grade scale (0–4 points), with a total score of 56. Higher scores indicate better balance. A score < 40 indicates a high risk of falls.
4. Walking ability
  Walking ability was assessed using the 10-Meter Walking Test (10MWT). Participants were instructed to walk at their fastest speed along a 16-m straight walkway. The start and end lines were marked with tape, and 3-m and 13-m points were marked with colored tape. Walking time from 3 m to 13 m was recorded and averaged over three trials. Walking speed was calculated in meters per second (m/s); higher values reflect better walking function.

### 2.5 Statistical analysis

All statistical analyses were carried out in SPSS 25.0. The Shapiro–Wilk test was used to check data normality, while Levene’s test was applied to test homogeneity of variance assumptions. Continuous variables are presented as mean ± standard deviation; categorical variables are expressed as n (%). Pearson χ^2^ test was adopted for between-group comparisons of categorical data. Two-way repeated-measures ANOVA (RMANOVA) was used to test main effects of group (conventional-rehabilitation, 3D-printed-AFO, combined-intervention), main effects of time (pre-intervention versus post-intervention), as well as group-by-time interaction effects. Cohen’s f was used for effect-size evaluation for RMANOVA results: trivial < 0.1, small 0.1–0.24, medium 0.25–0.39, large ≥ 0.4. Paired-samples t-test was applied for within-group pre-post comparisons. Tukey’s HSD was chosen for post-hoc pairwise comparison when variances were homogeneous; Tamhane’s T2 test was used when variance heterogeneity occurred. Bonferroni correction was used to reduce Type-I error inflation caused by multiple comparisons. Cohen’s d represented effect sizes for pairwise comparisons of change scores, with thresholds: small ≥ 0.2, medium ≥ 0.5, large ≥ 0.8.

For clinical responder analysis, because published anchor-based minimal clinically important difference (MCID) thresholds were unavailable for most included outcome measures in subacute-stroke older adults, all MCID values in the present study were derived using the 0.5-times-baseline-standard-deviation distribution-based method. Intra-class correlation coefficient (ICC) values for each measurement tool were extracted from previously published stroke-rehabilitation reliability studies. The minimal detectable change at the 95% confidence level (MDC₉₅) was calculated by the formula: MDC₉₅ = 1.96 × √2 × standard error of measurement (SEM), where SEM = SD × √(1-ICC). Each derived MCID threshold was explicitly checked against the corresponding MDC₉₅ value; all MCID thresholds exceeded MDC₉₅, ensuring that the defined improvement cut-offs were larger than random measurement error of the instruments. Subjects with pre-to-post intervention changes greater than or equal to the corresponding MCID were defined as clinical responders, and the Pearson χ^2^ test was used to compare responder rates among the three groups. It should be emphasized that responder-rate analysis was an exploratory post-hoc analysis and was not pre-specified in the trial registration document. The MCID thresholds for each indicator were as follows: improvement in active ankle dorsiflexion ≥ 4°, improvement in active ankle plantarflexion ≥ 7°; reduction in RMT ≥ 4%; increase in iEMG of target lower-limb muscles ≥ 15 μVs; decrease in CSI ≥ 2 points; increase in FMA-LE ≥ 3 points; increase in BBS ≥ 5 points; improvement in 10MWT walking speed ≥ 0.12 m/s. All statistical tests were two-tailed with a significance level of *α* = 0.05, and *p* < 0.05 was considered statistically significant.

## 3 Results

### 3.1 Baseline comparisons of participant characteristics

No significant inter-group differences were observed in baseline characteristics, including age, gender, stroke type, disease duration, and affected side among the three groups (all *p* > 0.05), indicating satisfactory baseline comparability (Table 1).

**Table 1:** Baseline characteristics of participants.

Variables	Conventional group (*n* = 20)	3D-printed group (*n* = 20)	Combined group (*n* = 20)	*F/ X^2^*	*p-*value
Age, mean ± SD	68.70 ± 5.89	68.20 ± 5.73	68.50 ± 5.38	0.025	0.975
Gender, *n* (%) Male/Female	12 (60%)/8 (40%)	11 (55%)/9 (45%)	7 (35%)/13 (65%)	2.800	0.247
Stroke type, *n* (%) Infarction/Hemorrhage	13 (65%)/7 (35%)	14 (70%)/6 (30%)	11 (55%)/9 (45%)	1.005	0.605
Disease duration (days), mean ± SD	68.00 ± 7.31	68.15 ± 7.65	68.00 ± 6.38	0.003	0.997
Affected side, *n* (%) Left/Right	12 (60%)/8 (40%)	7 (35%)/13 (65%)	10 (50%)/10 (50%)	2.536	0.281

### 3.2 Stratified analysis of outcome indicators

#### 3.2.1 Primary outcome measure: comparison of ankle active range of motion

Before intervention, there were no statistically significant differences in ankle dorsiflexion and plantarflexion range of motion among the three groups (*p* > 0.05). After 4-week intervention, the ankle dorsiflexion and plantarflexion range of motion displayed meaningful improvements in all three groups compared with baseline (*p* < 0.001) (Figure 2). RM-ANOVA revealed statistically significant main effects of group, main effects of time, and group-by-time interaction effects for both ankle dorsiflexion and plantarflexion range of motion (*p* < 0.05). Post-hoc multiple comparisons demonstrated that the improvement magnitudes of ankle dorsiflexion and plantarflexion range of motion in the combined group were significantly greater than those in the conventional group (*p* < 0.001) and the 3D-printing group (*p* = 0.022 for dorsiflexion, *p* = 0.005 for plantarflexion). The improvement magnitude of plantarflexion range of motion in the 3D-printing group was larger than that in the conventional group (*p* = 0.020). The minimal clinically important difference (MCID) was defined as ≥4° for dorsiflexion improvement and ≥7° for plantarflexion improvement. Responder rates were as follows: conventional group: dorsiflexion responder rate = 40% (8/20), plantarflexion responder rate = 35% (7/20); 3D-printed AFO group: dorsiflexion responder rate = 60% (12/20), plantarflexion responder rate = 65% (13/20); combined group: dorsiflexion responder rate = 90% (18/20), plantarflexion responder rate = 95% (19/20). The combined group exhibited a significantly higher responder rate compared with the conventional group and the 3D-printed AFO group (*p* < 0.05) (Table 2; Figures 3, 4).

**Figure 2:**
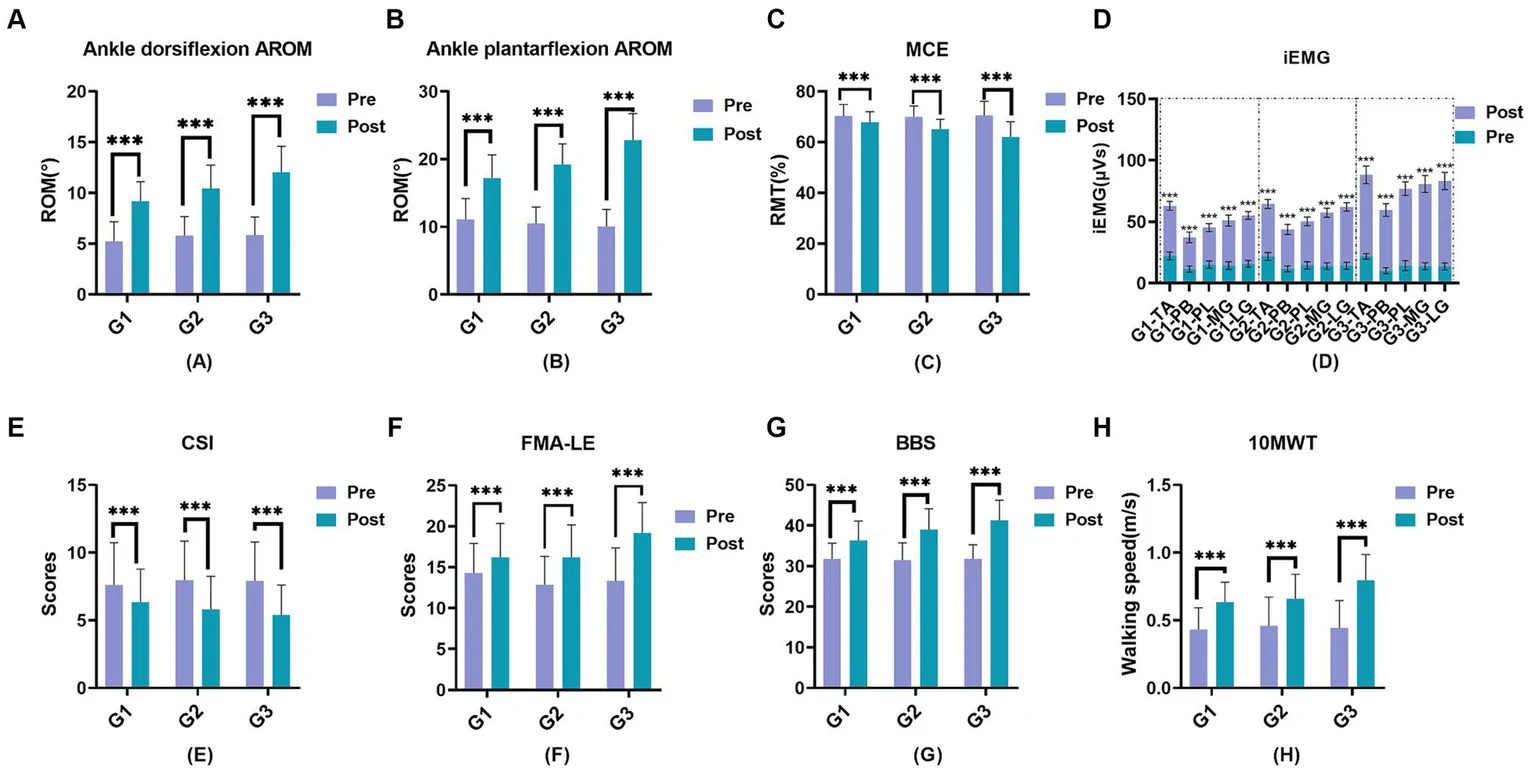
Comparisons of outcome measures before and after intervention in the three groups. **(A)** Active ankle dorsiflexion ROM. **(B)** Active ankle plantarflexion ROM. **(C)** Motor cortex excitability (MCE). **(D)** Integrated electromyography (iEMG) of the tibialis anterior (TA), peroneus brevis (PB), peroneus longus (PL), medial gastrocnemius (MG), and lateral gastrocnemius (LG). **(E)** Clinical Spasticity Index (CSI). **(F)** Fugl-Meyer Assessment-Lower Extremity (FMA-LE). **(G)** Berg Balance Scale (BBS). **(H)** 10-meter walking test (10MWT). G1: Conventional group; G2: 3D-printed group; G3: Combined group. ***, significant intergroup difference (*p* < 0.001).

**Table 2:** Comparison of active ankle dorsiflexion and plantarflexion ROM before and after intervention.

Outcome metric	Group	Pre (°) Mean ± SD	Post (°) Mean ± SD	Within-group *P*	Change Δ (Post-Pre)	95% CI of Δ	*P* vs. G1	*P* vs. G2	Cohen’s *d* vs. G1	Cohen’s *d* vs. G2
Ankle dorsiflexion	G1	5.25 ± 1.92	9.20 ± 1.91	<0.001	3.95 ± 1.32	[3.37, 4.53]	—	—	—	—
	G2	5.80 ± 1.88	10.45 ± 2.31	<0.001	4.65 ± 1.66	[3.87, 5.43]	0.437	—	0.47	—
	G3	5.85 ± 1.78	12.05 ± 2.54	<0.001	6.20 ± 2.26	[5.14, 7.26]	<0.001	0.022	1.22	0.78
Ankle plantarflexion	G1	11.10 ± 3.90	17.20 ± 3.43	<0.001	6.10 ± 3.06	[4.67, 7.53]	—	—	—	—
	G2	10.50 ± 2.42	19.20 ± 3.05	<0.001	8.70 ± 2.60	[7.49, 9.91]	0.020	—	0.92	—
	G3	10.05 ± 2.52	22.80 ± 3.94	<0.001	12.75 ± 3.18	[11.26, 14.24]	<0.001	0.005	2.13	1.40

Two-way repeated-measures ANOVA results: For ankle dorsiflexion: Main effect of group: F (2, 57) = 4.251, *p* = 0.019, Cohen’s f = 0.386; Main effect of time: F (1, 57) = 455.584, *p* < 0.001, Cohen’s f = 2.827; Interaction effect of group × time: F (2, 57) = 8.273, *p* < 0.001, Cohen’s f = 0.539. For ankle plantarflexion: Main effect of group: F (2, 57) = 3.598, *p* = 0.034, Cohen’s f = 0.355; Main effect of time: F (1, 57) = 579.449, p < 0.001, Cohen’s f = 3.188; Interaction effect of group × time: F (2, 57) = 25.722, *p* < 0.001, Cohen’s f = 0.950. G1: Conventional group; G2: 3D-printed group; G3: Combined group. Δ = post-intervention value - pre-intervention value; 95% CI represents the 95% confidence interval of the difference; Cohen’s d is the standardized effect size for comparing differences between groups.

**Figure 3:**
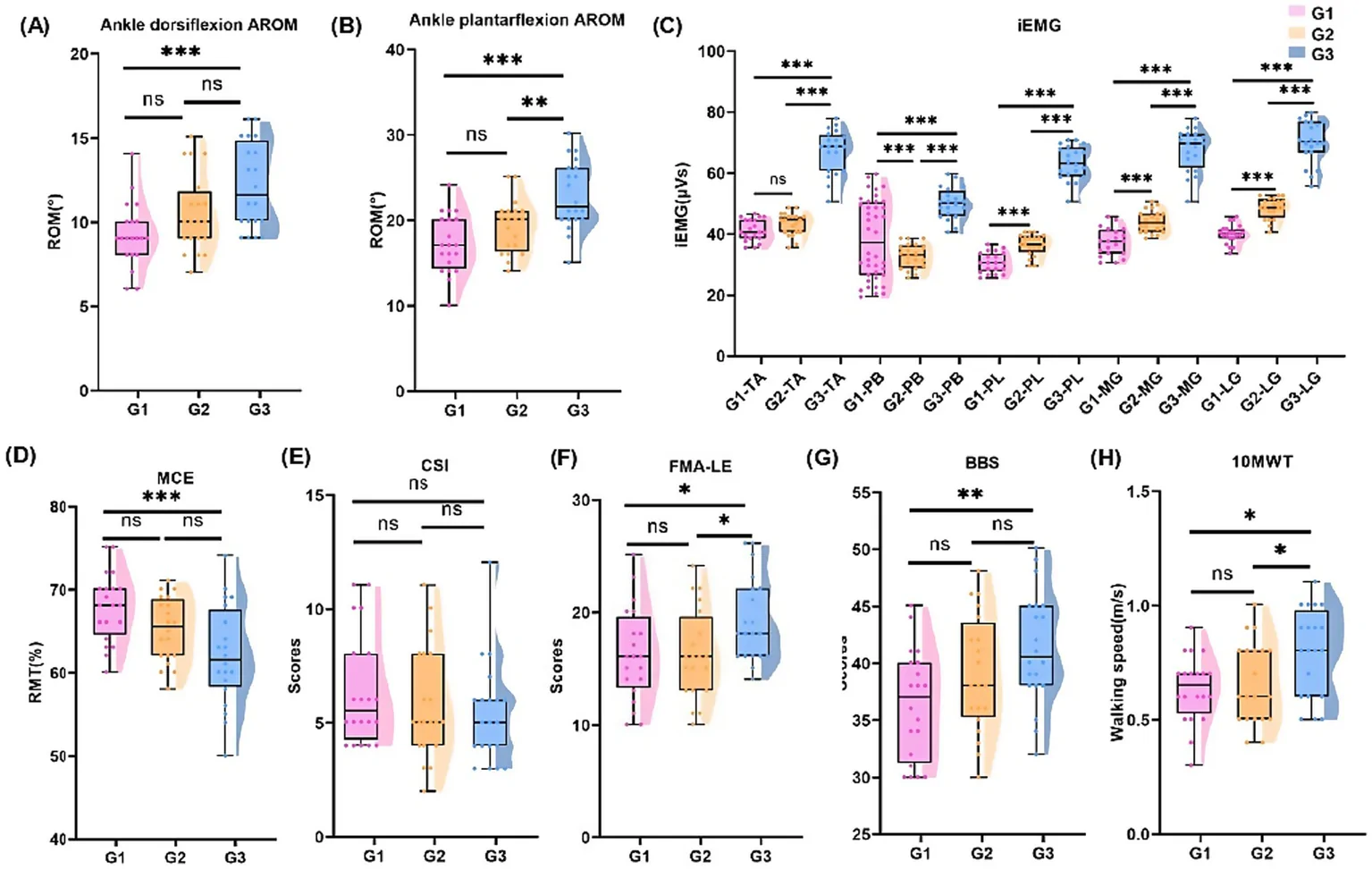
Post-intervention comparisons among the three groups (raincloud plots). **(A)** Active ankle dorsiflexion ROM. **(B)** Active ankle plantarflexion ROM. **(C)** MCE. **(D)** iEMG of TA, PB, PL, MG, and LG. **(E)** CSI. **(F)** FMA-LE. **(G)** BBS. **(H)** 10MWT. G1: Conventional group; G2: 3D-printed group; G3: Combined group. Data are presented as median (interquartile range, IQR) with individual data points overlaid. Raincloud plots illustrate data distribution: the box represents the 25th–75th percentiles, the horizontal line within the box denotes the median, and whiskers extend to the minimum and maximum values. ***, significant intergroup difference (*p* < 0.001); **, significant intergroup difference (*p* < 0.01); *, significant intergroup difference (*p* < 0.05); NS, non-significant intergroup difference (*p* > 0.05).

**Figure 4:**
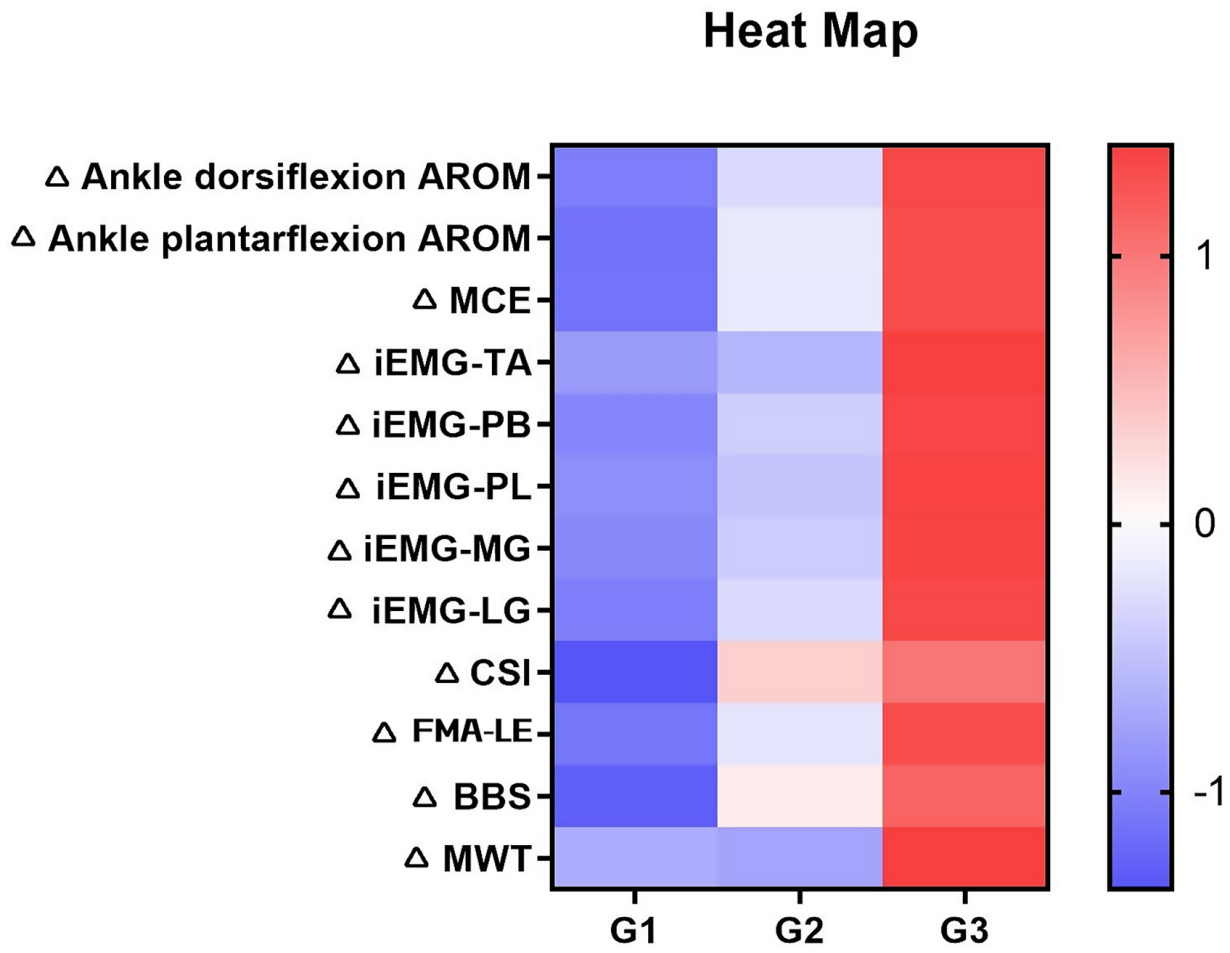
Heatmap of outcome changes (post-pre) in the three groups. Data were standardized using the Z-score. Blue to red indicates increasing improvement amplitude. G1: Conventional group; G2: 3D-printed group; G3: Combined group.

#### 3.2.2 Secondary electrophysiological mechanism outcomes

##### 3.2.2.1 Comparison of motor cortical excitability

Before intervention, no statistically significant differences in contralateral MCE were observed among the three groups at baseline (*p* > 0.05). After 4-week intervention, MCE was significantly decreased in all three groups relative to baseline (*p* < 0.001) (Figure 2). The results of RM-ANOVA showed that the main effect of time and the group-by-time interaction effect for MCE were statistically significant (*p* < 0.001), whereas the main effect of group was not statistically significant (*p* > 0.05). Post-hoc multiple comparisons indicated that the reduction magnitude of MCE in the combined group was significantly greater than that in the conventional group (*p* < 0.001) and the 3D-printed group (*p* < 0.001). The magnitude of reduction in MCE in the 3D-printed group was significantly greater than that in the conventional group (*p* = 0.002). The MCID was defined as an RMT reduction ≥ 4%. The responder rates were 20% (4/20) in the conventional group, 55% (11/20) in the 3D-printed group, and 90% (18/20) in the combined group. Significantly more patients exhibited improved cortical excitability in the combined group (*p* < 0.01) (Table 3; Figures 3, 4).

**Table 3:** Comparison of resting motor threshold (%) before and after intervention.

Group	Pre Mean ± SD	Post Mean ± SD	Within-group *P*	Change Δ (Post-Pre)	95% CI of Δ	*P* vs. G1	*P* vs. G2	Cohen’s *d* vs. G1	Cohen’s *d* vs. G2
G1	70.35 ± 4.51	67.85 ± 4.13	<0.001	−2.50 ± 1.73	[−3.31, −1.69]	—	—	—	—
G2	69.90 ± 4.35	65.10 ± 3.88	<0.001	−4.80 ± 2.14	[−5.80, −3.80]	0.002	—	1.18	—
G3	70.50 ± 5.61	62.05 ± 6.05	<0.001	−8.45 ± 2.28	[−9.52, −7.38]	<0.001	<0.001	2.94	1.65

Two-way repeated-measures ANOVA results for resting motor threshold: Main effect of group: F (2, 57) = 1.809, *p* = 0.173, Cohen’s f = 0.252; Main effect of time: F (1, 57) = 387.677, *p* < 0.001, Cohen’s f = 2.608; Interaction effect of group × time: F (2, 57) = 42.208, *p* < 0.001, Cohen’s f = 1.217. G1: Conventional group; G2: 3D-printed group; G3: Combined group. Δ = post-intervention value - pre-intervention value; 95% CI represents the 95% confidence interval of the difference; Cohen’s d is the standardized effect size for comparing differences between groups.

##### 3.2.2.2 Comparison of surface electromyography

At baseline, there were no statistically significant inter-group differences in the iEMG of the TA, PB, PL, MG and LG muscles among the three groups (*p* > 0.05). After 4-week intervention, the iEMG values of the above-mentioned five muscles were significantly increased in all three groups compared with baseline (all *p* < 0.001) (Figure 2). RM-ANOVA demonstrated statistically significant main effects of time, main effects of group, and group-by-time interaction effects for the iEMG of all five muscles (all *p* < 0.001). Post-hoc multiple comparisons revealed that the increased magnitudes of iEMG for each muscle in the combined group were significantly higher than those in the conventional group and the 3D-printed group (all *p* < 0.001). The increased iEMG magnitudes of PB, PL, MG and LG in the 3D-printed group were significantly greater than those in the conventional group (*p* < 0.01), whereas no significant difference was found in the increased iEMG magnitude of TA between the 3D-printed group and the conventional group (*p* > 0.05). Heterogeneity of variance was observed for the differences of MG and LG (Levene’s test, *p* < 0.05); therefore, Tamhane’s T2 test with Bonferroni correction was applied. The MCID was defined as iEMG increase ≥ 15 μVs. The responder rates for all muscles in the combined group were greater than 85%, which were significantly higher than those in the conventional group and the 3D-printed group (*p* < 0.05) (Table 4; Figures 3, 4).

**Table 4:** Comparison of iEMG (μVs) of lower limb muscles before and after intervention.

Muscle	Group	Pre Mean ± SD	Post Mean ± SD	Within-group *P*	Change Δ (Post-Pre)	95% CI of Δ	*P* vs. G1	*P* vs. G2	Cohen’s *d* vs. G1	Cohen’s *d* vs. G2
TA	G1	22.30 ± 3.15	40.65 ± 3.56	<0.001	18.35 ± 5.00	[16.01, 20.69]	—	—	—	—
	G2	21.85 ± 3.23	42.70 ± 3.69	<0.001	20.85 ± 5.70	[18.18, 23.52]	0.440	—	0.47	—
	G3	21.85 ± 2.23	66.40 ± 7.19	<0.001	44.55 ± 8.14	[40.74, 48.36]	<0.001	<0.001	3.88	3.37
PB	G1	11.55 ± 2.65	25.80 ± 4.37	<0.001	14.25 ± 5.66	[11.60, 16.90]	—	—	—	—
	G2	11.70 ± 2.43	32.15 ± 4.11	<0.001	20.45 ± 4.94	[18.14, 22.76]	0.001	—	1.17	—
	G3	10.20 ± 2.40	25.80 ± 4.37	<0.001	39.20 ± 5.13	[36.80, 41.60]	<0.001	<0.001	4.62	3.73
PL	G1	15.10 ± 2.96	30.15 ± 3.34	<0.001	15.05 ± 4.26	[13.06, 17.04]	—	—	—	—
	G2	14.50 ± 2.95	35.85 ± 3.50	<0.001	21.35 ± 5.32	[18.86, 23.84]	0.002	—	1.31	—
	G3	14.30 ± 4.00	62.65 ± 5.56	<0.001	48.35 ± 6.82	[45.16, 51.54]	<0.001	<0.001	5.86	4.42
MG	G1	14.30 ± 3.08	36.75 ± 4.52	<0.001	22.45 ± 5.80	[19.74, 25.16]	—	—	—	—
	G2	13.95 ± 2.72	43.45 ± 3.66	<0.001	29.50 ± 3.97	[27.64, 31.36]	<0.001	—	1.42	—
	G3	13.90 ± 2.81	66.90 ± 6.87	<0.001	53.00 ± 8.16	[49.18, 56.82]	<0.001	<0.001	4.32	3.66
LG	G1	15.80 ± 2.75	39.40 ± 3.10	<0.001	23.60 ± 3.08	[22.16, 25.04]	—	—	—	—
	G2	14.25 ± 2.79	47.85 ± 3.47	<0.001	33.60 ± 4.65	[31.42, 35.78]	<0.001	—	2.53	—
	G3	13.70 ± 2.89	69.55 ± 7.06	<0.001	55.85 ± 7.87	[52.17, 59.53]	<0.001	<0.001	5.40	3.44

Two-way repeated-measures ANOVA results: TA: Main effect of group: F (2, 57) = 145.226, *p* < 0.001, Cohen’s f = 2.257; Main effect of time: F (1, 57) = 1134.165, *p* < 0.001, Cohen’s f = 4.461; Interaction effect of group × time: F (2, 57) = 101.416, p < 0.001, Cohen’s f = 1.886. PB: Main effect of group: F (2, 57) = 98.567, *p* < 0.001, Cohen’s f = 1.860; Main effect of time: F (1, 57) = 1320.224, *p* < 0.001, Cohen’s f = 4.813; Interaction effect of group × time: F (2, 57) = 122.384, *p* < 0.001, Cohen’s f = 2.072. PL: Main effect of group: F (2, 57) = 210.756, *p* < 0.001, Cohen’s f = 2.719; Main effect of time: F (1, 57) = 1545.468, *p* < 0.001, Cohen’s f = 5.207; Interaction effect of group × time: F (2, 57) = 210.999, *p* < 0.001, Cohen’s f = 2.662. MG: Main effect of group: F (2, 57) = 153.689, *p* < 0.001, Cohen’s f = 2.322; Main effect of time: F (1, 57) = 1900.820, *p* < 0.001, Cohen’s f = 5.775; Interaction effect of group × time: F (2, 57) = 132.473, *p* < 0.001, Cohen’s f = 2.156. LG: Main effect of group: F (2, 57) = 131.869, *p* < 0.001, Cohen’s f = 2.151; Main effect of time: F (1, 57) = 2746.665, *p* < 0.001, Cohen’s f = 6.942; Interaction effect of group × time: F (2, 57) = 175.706, *p* < 0.001, Cohen’s f = 2.483. G1: Conventional group; G2: 3D-printed group; G3: Combined group. TA: tibialis anterior; PB: peroneus brevis; PL: peroneus longus; MG: medial gastrocnemius; LG: lateral gastrocnemius. Δ = post-intervention value - pre-intervention value; 95% CI represents the 95% confidence interval of the difference; Cohen’s d is the standardized effect size for comparing differences between groups.

#### 3.2.3 Secondary clinical functional outcomes

##### 3.2.3.1 Comparison of CSI, FMA-LE and BBS scores

At baseline, there were no statistically significant differences in the CSI, FMA-LE and BBS scores among the three groups (*p* > 0.05). After the 4-week intervention, the CSI scores were significantly decreased, whereas the FMA-LE and BBS scores were significantly increased in all three groups compared with baseline (all *p* < 0.001) (Figure 2). RM-ANOVA revealed statistically significant main effects of time and group-by-time interaction effects for the three scores (all *p* < 0.01), while no significant main effect of group was observed (all *p* > 0.05). Post-hoc multiple comparisons showed the following results. For CSI: the reduction magnitudes in the combined group and the 3D-printed group were significantly greater than those in the conventional group (*p* < 0.05), and no significant difference was found between the combined group and the 3D-printed group (*p* > 0.05). For FMA-LE: the improvement magnitude in the combined group was significantly higher than that in the conventional group (*p* < 0.001), with no significant difference between the 3D-printed group and the conventional group (*p* > 0.05). For BBS: the improvement magnitudes in the combined group and the 3D-printed group were significantly greater than those in the conventional group (*p* < 0.01), and there was no significant difference between the combined group and the 3D-printed group (*p* > 0.05). The MCID thresholds were defined as: CSI reduction ≥ 2 scores; FMA-LE increase ≥ 3 scores; BBS increase ≥ 5 scores. For CSI responder rate: 30% (6/20) in the conventional group, 65% (13/20) in the 3D-printed group, and 70% (14/20) in the combined group. No significant difference was detected between the combined group and the 3D-printed group (*p* > 0.05), whereas both groups exhibited superior responder rates relative to the conventional group (*p* < 0.05). For FMA-LE responder rate: 35% (7/20) in the conventional group, 60% (12/20) in the 3D-printed group, and 90% (18/20) in the combined group, with statistically significant inter-group differences (*p* < 0.05). For BBS responder rate: 40% (8/20) in the conventional group, 70% (14/20) in the 3D-printed group, and 95% (19/20) in the combined group. The combined group yielded a significantly higher clinical responder rate (*p* < 0.05). See Table 5 for details (Figures 3, 4).

**Table 5:** Comparison of CSI, FMA-LE and BBS scores before and after intervention.

Indicator	Group	Pre Mean ± SD	Post Mean ± SD	Within-group *P*	Change Δ (Post-Pre)	95% CI of Δ	*P* vs. G1	*P* vs. G2	Cohen’s *d* vs. G1	Cohen’s *d* vs. G2
CSI	G1	7.60 ± 3.14	6.35 ± 2.43	<0.001	−1.25 ± 1.02	[−1.73, −0.77]	—	—	—	—
	G2	7.95 ± 2.89	5.80 ± 2.44	<0.001	−2.15 ± 1.09	[−2.66, −1.64]	0.046	—	0.85	—
	G3	7.90 ± 2.88	5.40 ± 2.21	<0.001	−2.50 ± 1.35	[−3.12, −1.86]	0.004	0.611	1.04	0.28
FMA-LE	G1	14.30 ± 3.61	16.20 ± 4.15	<0.001	1.90 ± 1.71	[1.10, 2.70]	—	—	—	—
	G2	12.85 ± 3.47	16.20 ± 3.96	<0.001	3.35 ± 2.37	[2.24, 4.46]	0.058	—	0.70	—
	G3	13.35 ± 4.02	19.20 ± 3.71	<0.001	5.85 ± 1.73	[5.04, 6.66]	<0.001	0.050	2.30	1.21
BBS	G1	31.75 ± 3.91	36.30 ± 4.81	<0.001	4.55 ± 3.35	[2.98, 6.12]	—	—	—	—
	G2	31.50 ± 4.22	39.00 ± 5.13	<0.001	7.50 ± 2.86	[6.16, 8.84]	0.009	—	0.95	—
	G3	31.75 ± 3.54	41.30 ± 4.93	<0.001	9.55 ± 2.86	[8.21, 10.89]	<0.001	0.091	1.61	0.72

Two-way repeated-measures ANOVA results: CSI: Main effect of group: F (2, 57) = 0.081, *p* = 0.923, Cohen’s f = 0.053; Main effect of time: F (1, 57) = 171.123, *p* < 0.001, Cohen’s f = 1.733; Interaction effect of group × time: F (2, 57) = 6.133, *p* = 0.004, Cohen’s f = 0.464. FMA-LE: Main effect of group: F (2, 57) = 1.130, *p* = 0.330, Cohen’s f = 0.199; Main effect of time: F (1,57) = 213.887, *p* < 0.001, Cohen’s f = 1.937; Interaction effect of group × time: F (2, 57) = 20.792, *p* < 0.001, Cohen’s f = 0.854. BBS: Main effect of group: F (2, 57) = 1.775, *p* = 0.179, Cohen’s f = 0.250; Main effect of time: F (1, 57) = 339.057, *p* < 0.001, Cohen’s f = 2.439; Interaction effect of group × time: F (2, 57) = 13.773, *p* < 0.001, Cohen’s f = 0.695. G1: Conventional group; G2: 3D-printed group; G3: Combined group. Δ = post-intervention value - pre-intervention value; 95% CI represents the 95% confidence interval of the difference; Cohen’s d is the standardized effect size for comparing differences between groups.

##### 3.2.3.2 Comparison of 10MWT walking speed

At baseline, there was no statistically significant inter-group difference in 10MWT among the three groups (*p* > 0.05). After 4-week intervention, 10MWT was significantly improved in all three groups compared with baseline (*p* < 0.001) (Figure 2). RM-ANOVA showed statistically significant main effects of time and group-by-time interaction effect for 10MWT (*p* < 0.05), whereas the main effect of group was not statistically significant (*p* > 0.05). Post-hoc multiple comparisons revealed that the improvement magnitude of 10MWT in the combined group was significantly greater than that in the conventional group (*p* = 0.013) and the 3D-printed group (*p* = 0.024). At the same time, no significant difference was observed between the 3D-printed group and the conventional group (*p* > 0.05). The MCID was defined as a walking speed increase ≥ 0.12 m/s. The 10MWT responder rates were 30% (6/20) in the conventional group, 55% (11/20) in the 3D-printed group, and 90% (18/20) in the combined group, with a statistically significant difference among groups (*p* < 0.05) (Table 6; Figures 3, 4).

**Table 6:** Comparison of 10MWT (m/s) before and after intervention.

Group	Pre Mean ± SD	Post Mean ± SD	Within-group *P*	Change Δ (Post-Pre)	95% CI of Δ	*P* vs. G1	*P* vs. G2	Cohen’s *d* vs. G1	Cohen’s *d* vs. G2
G1	0.43 ± 0.16	0.63 ± 0.15	<0.001	0.21 ± 0.22	[0.10, 0.31]	—	—	—	—
G2	0.46 ± 0.21	0.66 ± 0.18	<0.001	0.20 ± 0.15	[0.13, 0.27]	0.996	—	−0.03	—
G3	0.45 ± 0.20	0.80 ± 0.19	<0.001	0.35 ± 0.15	[0.28, 0.42]	0.013	0.024	0.78	1.00

Two-way repeated-measures ANOVA results for 10-Metre Walking Test: Main effect of group: F (2, 57) = 1.551, *p* = 0.221, Cohen’s f = 0.233; Main effect of time: F (1, 57) = 123.109, *p* < 0.001, Cohen’s f = 1.470; Interaction effect of group × time: F (2, 57) = 4.703, *p* = 0.013, Cohen’s f = 0.406. G1: Conventional group; G2: 3D-printed group; G3: Combined group. Δ = post-intervention value - pre-intervention value; 95% CI represents the 95% confidence interval of the difference; Cohen’s d is the standardized effect size for comparing differences between groups.

## 4 Discussion

In this investigation, we contrasted conventional rehabilitation, 3D-printed AFO plus sham rTMS, and 3D-printed AFO plus active high-frequency rTMS among older patients suffering from post-stroke ankle-foot dysfunction in the subacute stage (21). All three intervention arms brought meaningful improvements in ankle range of motion, corticospinal excitability-related indicators, peri-ankle muscle activation, spasticity, balance and walking performance. Even so, participants receiving combined therapy achieved larger functional gains and higher clinical responder rates for most measured endpoints. It should be stressed that RMT and iEMG are merely indirect neurophysiological surrogate markers. Our three-arm trial did not set up an rTMS-only comparison group; for this reason, additive therapeutic effects cannot be statistically separated from potential synergistic interactions. In the following sections, we explore possible explanations for better outcomes in the combined-therapy group, propose exploratory, hypothesis-generating considerations for future patient-stratification research, and point out major inherent limitations of this work.

### 4.1 Analysis of reasons for the superior efficacy of the combined group

Several meta-analyses indicate that rTMS improves ankle dorsiflexion, lower limb motor function (22), and walking speed after stroke (23). These systematic reviews indicate that rTMS modulates cortical excitability (24) and strengthens descending corticospinal drive when applied over the primary motor cortex (25). However, nearly all previous studies only focused on rTMS monotherapy or rTMS combined with conventional training, and did not use personalized 3D-printed AFO to augment peripheral orthotic support.

Conventional rehabilitation helps increase joint range of motion, muscle strength, and walking ability, but it does little to drive central neuroplasticity or provide precise mechanical correction. As a result, functional gains are often limited and recovery takes longer. High-frequency rTMS applied to the primary motor cortex (M1 area) can shorten reflex motor latency, enhance cortical excitability, improve central drive to the ankle-foot muscle groups, thereby optimizing the coordination of lower limb muscle contraction and relieving spasticity (18). In this study, decreased RMT and increased iEMG were observed in the combined group. These observations point toward a link between rTMS intervention, elevated corticospinal excitability and better muscle-activation status. Still, these two markers serve only as indirect surrogates and cannot offer direct evidence for neuroplastic remodeling processes (12). However, rTMS alone cannot mechanically correct persistent biomechanical abnormalities such as foot drop and varus deformity.

Using 3D scanning, digital modeling, and rapid prototyping, a 3D-printed AFO can correct ankle-foot deformities more directly than conventional orthoses (26). In our experience, custom-made devices fit better, cause fewer skin problems, and may lead to better rehabilitation outcomes. The orthosis improves the mechanical conditions during both stance and swing. It also reduces compensatory movements and fall risk, while enhancing proprioceptive input from the periphery to the central nervous system. It remains a theoretical conjecture that proprioceptive input promotes neural-circuit remodeling; we did not carry out direct measurement of proprioceptive signals in this trial. The results of this study showed that the 3D-printed group was superior to the conventional rehabilitation group in active ankle range of motion (AROM), Clinical Spasticity Index (CSI), Berg Balance Scale (BBS), and 10-meter walking test (10MWT).

The 3D-printed-AFO-only intervention brought optimized peripheral biomechanical correction and increased proprioceptive sensory input, yet it could not independently raise descending efferent motor output originating from the lesioned cortex. We raise an unvalidated exploratory speculation that combining these two treatment modalities may build a neuromechanical closed-loop rehabilitation pathway. Nevertheless, our dataset supplies no direct empirical evidence for such closed-loop effects. Because our three-arm RCT lacks a separate rTMS-only control arm, we cannot statistically tell apart simple additive therapeutic benefits and potential synergistic interactions between central rTMS neuromodulation and peripheral orthotic support. rTMS elevates cortical excitability at the target brain region and transmits motor signals; the AFO ensures movement accuracy and gait stability by correcting gait patterns; continuous proprioceptive feedback may further promote cortical plasticity and progressively improve motor control (Figure 5).

**Figure 5:**
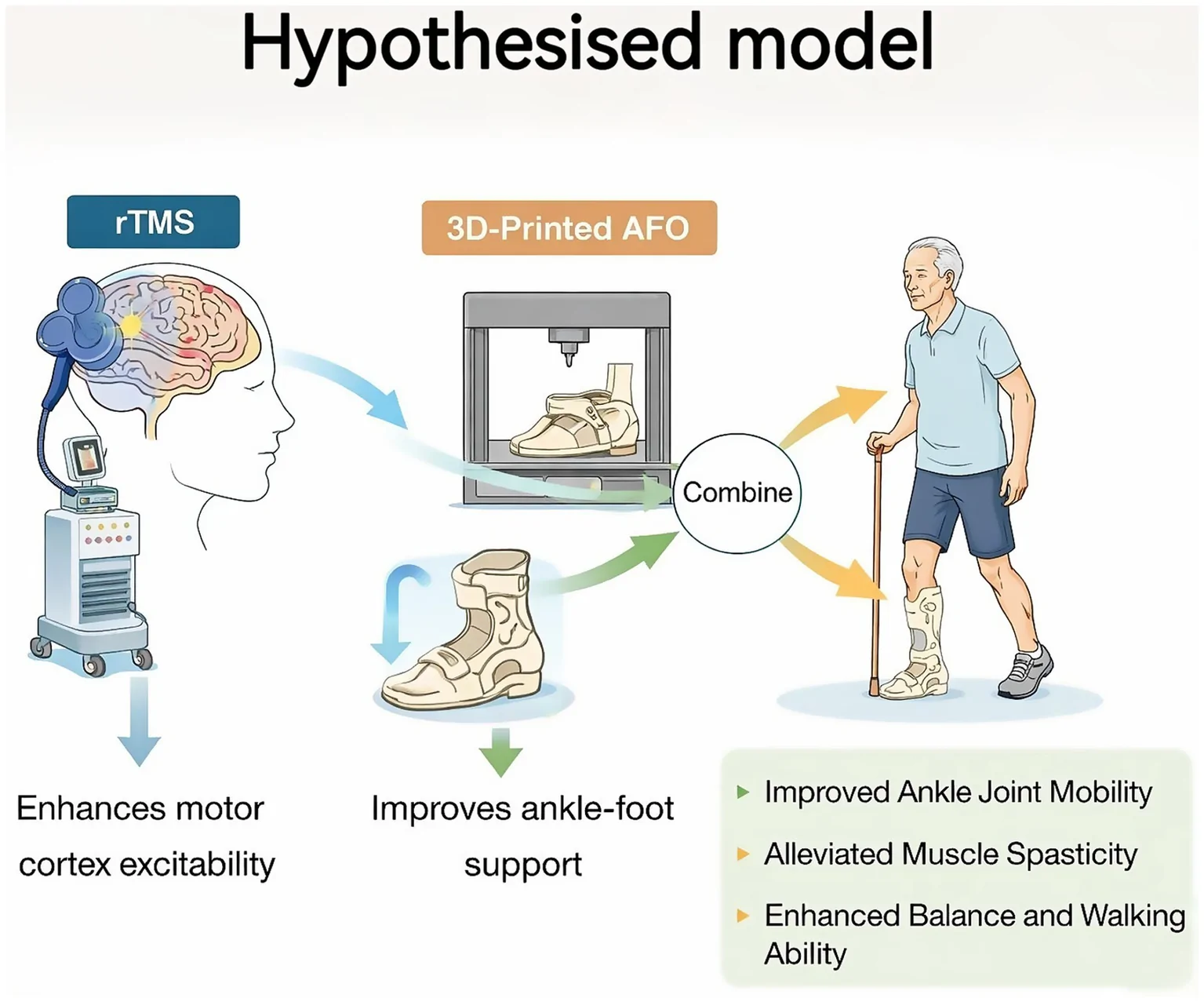
Hypothesized mechanistic pathways explaining incremental functional gains under combined rTMS plus 3D-printed AFO intervention. This schematic represents an exploratory model derived purely from surrogate physiological markers and clinical outcome data collected in this trial. According to this speculative framework, high-frequency rTMS elevates corticospinal excitability within the lesioned primary motor cortex and augments descending motor output. The 3D-printed AFO delivers mechanical orthotic correction and peripheral proprioceptive afferent stimulation. Augmented descending motor signals combined with orthotic mechanical support may enhance muscle recruitment and functional motor performance. Importantly, the present trial contained no direct assays of proprioceptive afferent traffic or neural-circuit reorganization; therefore, this proposed pathway cannot be empirically validated here and requires dedicated future investigations.

The reasoning outlined above represents a hypothesis built purely on observational data collected in this study. Better outcomes within the combined group may come from simple additive benefits from the two therapies, or alternatively from mutual synergistic effects; our present trial design cannot differentiate these two possibilities.

### 4.2 Hypothesized mechanisms for the combined-group benefits: sensorimotor integration and neuroplastic feedback

Biological mechanisms responsible for greater functional improvements under combined treatment are not fully understood. Based on our clinical and neurophysiological observations, we put forward an exploratory hypothesis: combining rTMS neuromodulation and 3D-printed orthotic support may improve afferent-efferent signaling-pathway function. We would highlight that our dataset contains no direct evidence confirming recovery or structural reorganization of these neural circuits. Regarding specific outcomes, the improvement in active ankle plantarflexion ROM in the combined group was significantly better than that in the 3D-printed AFO group, whereas no significant difference was found in active dorsiflexion improvement between the two groups. These results suggest that the mechanical corrective effect of the 3D-printed AFO on dorsiflexion approaches a plateau. The addition of rTMS may specifically increase neural efferent output to the tibialis anterior, peroneal muscle group, and gastrocnemius, thereby improving plantar flexion function (27). This reading stays speculative and is not backed up by direct neural-signal measurements. iEMG tells us about muscle contraction over time. In stroke patients with foot drop, iEMG values are usually low, and tracking this change helps assess recovery (28). The results of this study showed that the iEMG values of the tibialis anterior, peroneus brevis, peroneus longus, medial gastrocnemius, and lateral gastrocnemius in the combined group were significantly higher than those in the conventional rehabilitation group and the 3D-printed AFO group after intervention, suggesting that the combination may reduce the delayed muscle response often seen in post-stroke foot drop.

For spasticity relief, both the combined group and the 3D-printed AFO group were superior to the conventional group, with no significant difference between the former two groups, suggesting that external mechanical stretching and support are crucial for reducing lower limb muscle spasticity. The 3D-printed AFO optimizes lower limb biomechanical alignment, reduces compensatory movements (13), and provides continuous proprioceptive afferent feedback to the sensorimotor cortex. How proprioceptive feedback acts upon the cerebral cortex remains a hypothesis in this work and has not been validated via direct measurements.

The combined group showed the most prominent improvements in lower limb motor function (FMA-LE), balance function (BBS), and walking speed (10MWT). During lower limb movement, integrated proprioceptive afferent signals promote neuroplastic changes, allowing the central nervous system to generate more accurate and efficient motor commands. In the present study, we observed that rTMS reduced RMT (indicating increased corticospinal excitability at the target side), and AFO provided stable peripheral input (29). Even so, we cannot directly capture proprioceptive afferent signals or sensorimotor-integration processes. Therefore, the synergistic motor-control improvement produced by the two interventions remains hypothetical. This hypothesis could explain improvements in patients’ lower-limb motor control, balance, and walking ability, but dedicated trials are still required to verify it.

The conventional group received neither central neural modulation nor customized mechanical support, thereby obtaining the minimal rehabilitation benefits. Although the 3D-printed AFO group achieved better outcomes than the conventional group, the absence of rTMS-mediated central modulation failed to verify the optimization of the afferent-efferent loop in this study. Only limited alterations in cortical excitability and marginal functional improvements were observed in this group.

### 4.3 Hypothesized considerations for future patient-stratification research on the combined intervention

Since formal, prespecified subgroup analyses were not performed in this trial, the following analyses are exploratory in nature. They are intended to inform the design of future patient-stratification studies and should not be regarded as validated clinical indications or established treatment pathways.

Our preliminary clinical observations suggest that the combined intervention warrants dedicated investigation in patients with persistent foot drop or varus gait following stroke. In routine clinical practice, such patients typically present with a lower-extremity Brunnstrom stage ≥ 3, active ankle dorsiflexion < 10°, mild-to-moderate spasticity (Clinical Spasticity Index 0–12), the ability to walk ≥ 10 m with or without supervision, a disease duration of 1–6 months, and reduced or absent proprioception. It should be emphasized that these characteristics represent only descriptive clinical observations from our setting and are not validated predictors of treatment response. No conclusions can be drawn from the present study regarding whether additional benefit can be achieved by combined therapy in patients with mild ankle-foot dysfunction who respond well to conventional rehabilitation. Among the elderly patients in our sample who had persistent foot drop, unstable gait, or sensory impairments, the combined intervention appeared to yield more efficient rehabilitation outcomes. Nevertheless, this remains an unconfirmed clinical observation and does not constitute a definitive treatment recommendation.

Future, adequately powered patient-stratification studies may consider stratifying by stroke type (cerebral infarction / intracerebral hemorrhage), lesion location and affected side, Brunnstrom stage, baseline proprioceptive function (normal / impaired / absent), disease course (subacute / convalescent phase), and foot-drop severity (mild / moderate). Such stratification would help to refine potential indications for combining rTMS with a 3D-printed AFO, pending validation in dedicated trials.

### 4.4 Limitations and future directions

This study has several limitations. First, it is a single-center trial with a limited sample size; generalizability of findings should be interpreted with caution. Second, the intervention lasted only 4 weeks without long-term follow-up. The durability of functional gains and optimal treatment duration remain unknown. Third, this three-arm RCT lacked an rTMS-only group, and a four-arm factorial design was not feasible. We cannot distinguish additive from synergistic effects and can only confirm incremental benefits of adding rTMS to 3D-AFO. Future four-arm multicenter trials are needed to disentangle individual and interactive effects of central neuromodulation and peripheral orthosis. Fourth, RMT and iEMG are indirect surrogate markers. RMT only reflects corticospinal excitability of the ipsilesional primary motor cortex, while iEMG represents only peripheral peri-ankle muscle activation. Neither can directly measure proprioceptive afferent signals, sensorimotor integration or neural circuit reorganization. Interpretations regarding afferent-efferent loops and neuromechanical closed-loop effects are exploratory hypotheses, requiring further validation using direct approaches such as functional MRI and quantitative proprioception assessment. Fifth, prespecified subgroup analyses were not performed, so subgroups with maximum benefit cannot be identified. Future stratified analyses by stroke subtype, lesion location, Brunnstrom stage, baseline proprioception, disease course and foot-drop severity will help refine clinical indications for this combined intervention.

Future work may integrate wearable sensors, electrophysiological monitoring and artificial intelligence to build an adaptive closed-loop rehabilitation system for real-time individualized adjustment of rTMS and AFO settings.

## 5 Conclusion

For older adults suffering from ankle-foot dysfunction in the subacute stroke phase, adding high-frequency rTMS to rehabilitation supported by 3D-printed AFO yielded greater clinical functional improvements compared with single-modality interventions, covering ankle mobility, corticospinal excitability metrics, muscle activation, spasticity indicators, balance and walking-speed endpoints. Considering RMT and iEMG act merely as indirect physiological surrogate markers, mechanistic explanations for sensorimotor integration and afferent-efferent-loop modulation built upon our findings stay exploratory hypotheses and need further empirical testing. Our results suggest that combining central neuromodulation and custom-tailored peripheral orthotic mechanical support carries encouraging translational potential for stroke-rehabilitation clinical work.

## Data Availability

The raw data supporting the conclusions of this article will be made available by the authors, without undue reservation.
